# A proposal to improve calibration and outlier detection in high-throughput mass spectrometry

**DOI:** 10.1016/j.clinms.2016.12.003

**Published:** 2017-01-03

**Authors:** Adam P.R. Zabell, Fred E. Lytle, Randall K. Julian

**Affiliations:** Indigo BioAutomation, 7820 Innovation Blvd, Suite 250, Indianapolis, IN 46278, United States

**Keywords:** Calibration, Outlier detection, Studentized deleted residual, Instrument stability

## Abstract

Instrument calibration, required for any accurate quantitative calculation, is a trivial process when performed correctly, but is also full of easily overlooked stumbling blocks. To minimize the risk of error associated with improper calibrations, national and international guidance dictates a minimum number of calibrators and the threshold at which a measurement becomes an outlier. Evidence from industry practice, which conflicts with regulatory guidance, suggests that most groups are focused on remapping their detector with each run. We present a *post facto* explanation for the calibrator minimum and provide recommendations for curve building, which include improved outlier detection for high-volume mass spectrometry laboratories.

## Highlights

• Seven calibration points implies a 95% confidence limit on the regression.
• Most labs use calibration to map the detector instead of confirm instrument stability.
• Quadratic or cubic fits to the data increase the risk of a poorly fit model.
• Serial dilution creates a leverage problem for the upper end of the range.

## 1 Introduction

Calibration curves are at the core of analytical science. However, their ubiquitous presence has resulted in an environment of casual implementation that tends to neglect consideration of the problem being addressed [1], [2]. A calibration curve should either confirm the analyte–response relationship or indicate the presence of an issue with the analytical technique. Critical to the curve’s success is an understanding of the limitations of the assay, the instrument, and the statistics used to build that curve. While a great deal of study goes into the selection of an instrument [3], [4], [5] and an analyte [6], [7], the choices behind curve calculation are regularly neglected. The work of Raposo [2] is one of the rare instances where this problem has been addressed. That lack of attention is unfortunate since no amount of statistical analysis, improved instrumentation, or replicate measurement can rescue good data from a bad calibration curve. Without appropriate design, issues will remain masked and outlier measurements will skew results.

Modern methods using liquid chromatography–tandem mass spectrometry (LC–MS/MS) allow biologically active organics, metabolites and proteins to be routinely quantified through a combination of internal standards and ion ratios [8], [9], [10], [11]. In the context of high-throughput quantification, each sample should be processed quickly and marked with a reported value, or a reason for resampling (*e.g.*, dilution required, flawed internal standard, incorrect ion ratio). Knowing the analyte concentration in the samples, which build the calibration curve, allows additional checks against specific quality expectations. Regulatory guidance provides a minimally required degree of quality in those samples, but is proscriptive without explanation for those limits. For example, draft guidance from the USFDA mandates a minimum of seven calibrants – six plus a zero concentration standard – to perform the calibration, but does not explain why the curve should have seven points instead of a minimum of four or ten [12], [13].

Industrial and governmental guidance for calibration varies. The three most relevant to the clinical and pre-clinical markets are the European Medicines Agency guideline on bioanalytical methods, issued in 2011[14], the Eurachem guide for method validation, issued in 2014 [15], and the draft industry guidance by the USFDA for bioanalytical method validation, issued in 2013 [12]. A summary of these three sources is provided in Table 1. Some approximations have been made in order to simplify the table, most notable being the distinction between a calibration curve built for initial testing, and a calibration curve used for instrument verification. Indeed, verification of response stability over time is the principal difference between traditional and regulated calibration.

**Table 1:** Basic calibration curve criteria.

Criteria	EMEA [14]	Eurachem [15]	USFDA (proposed) [12]
Minimum number of points	6	7	7
Blank included as one of those points	No	Yes	Yes
Replicates during analytic run	Not required	Duplicate	Not required
Working Range	From the LLOQ to ULOQ	The linear response range	From the LLOQ to ULOQ
Calibrant Spacing	Not mentioned	Evenly spaced	Not mentioned
Calibrant Accuracy	LLOQ: within 20% of nominal All Others: within 15% of nominal	Mentioned, but no limit specified	LLOQ: within 20% of nominal All Others: within 15% of nominal
Calibrant Precision	Not mentioned	3× the standard deviation (s.d.)	LLOQ: CV ⩽ 20% All Others: CV ⩽ 15%
Drop points for Calibration Curve	Yes, provided 75% of the standards are retained, with a minimum of six retained	Yes, but only after checking the measurements at nearby concentrations	Yes, provided 75% of collected points meet accuracy and precision, including the LLOQ
QC samples	6 total	5% of the total number of samples being run	5% of the number of unknowns, or 6 total, whichever is greater
QC replicates	2x at each level	Not required	2x at each level
QC acceptance	50% at each level are within 15% of nominal, and 67% of all collected are within 15% of nominal	‘warning limit’ at 2× s.d. and ‘action limit’ at 3× s.d.	50% at each level are within 15% of nominal, and 67% of all collected are within 15% of nominal
Permitted to extrapolate an unknown outside the working range	No	No	No

After verifying assay suitability, the concern becomes when and how to eliminate outlier effects. In practice, there are two methods employed to avoid this risk: increase the curve complexity or exclude observed outliers. Industrial practice permits significant latitude in deciding the curve fit and weight on a per run basis, seemingly independent of the method used during validation. Outlier exclusion is less often used, even though regulatory guidance provides simple and explicit rules about detection and removal.

Here, we present a framework for building a calibration curve and resolving outliers that result from establishment of the curve. First, we provide a general case argument for the minimum number of calibrants required by regulatory guidance. Then, we examine the poor success rate of simple outlier detection in calibration curves using either equidistant or logarithmic calibrant spacing, as well as the significant risks associated with extending the curve outside of the linear response region. Finally, we outline a statistically robust method that significantly improves the rate of outlier detection while maintaining guidance requirements. Our proposal calls for an alternative approach to LC–MS/MS calibration: run a dedicated standards batch and apply it to subsequent analytic runs until the quality control samples in that run exceed the proscribed tolerance. This method retains a check on analytic run accuracy without sacrificing samples to manual manipulation, rejecting a whole run because of a single outlier, or reducing the number of available samples on the well plate.

## 2 Theory and methods

### 2.1 Calibration definitions

**Scheme 1:**
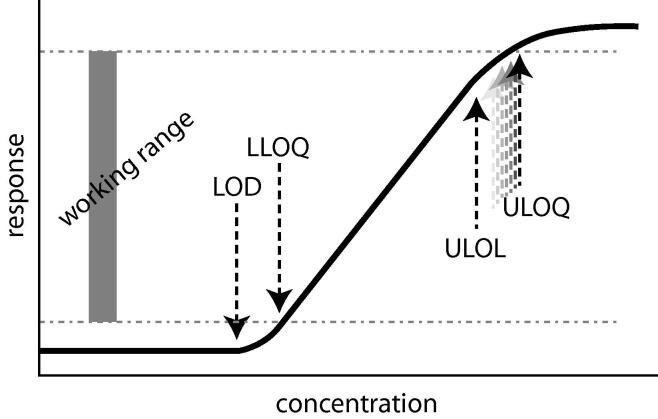
Depiction of the sigmoidal LC–MS/MS response curve, with labels for limit of detection (LOD), the lower limit of quantitation (LLOQ), the upper limit of linearity (ULOL), the upper limit of quantitation (ULOQ), and working range. The working range is defined from LLOQ to ULOQ, and the figure is drawn with the ULOQ permitted to extend past the linear portion of the response curve.

For LC–MS/MS data, the generalized sigmoidal response curve is shown in Scheme 1. At the lowest concentrations, the observable peak is either absent or within the noise level of the detector. At the highest concentrations, the detector is saturated and an observed response is no longer peak-like. Between these two extremities is a linear region of response to concentration. Ideally, the working range will be confined to this linear region, but there are situations where that range will be extended. Small molecule metabolites such as tenamphetamine are well-behaved into the upper nonlinear range, for example, while protein-digested peptides are often quantified through the lower nonlinear range.

Any comparison of known concentration to instrument response could be considered a calibration curve, but here we choose to explicitly distinguish between how these curves are built, according to the intended purpose of the regression.

**Type 1: Map the detector**. A series of standards is run across several orders of magnitude to check reproducibility at the limit of detection (LOD), the lower limit of quantitation (LLOQ), the upper limit of linearity (ULOL), the upper limit of quantitation (ULOQ), and set intervals between LLOQ and ULOQ. The working range for that analyte is defined as the response between the LLOQ and ULOQ. When the calibration curve is not linear, calibrants should be clustered at the inflection points where the curve deviates from linearity in order to verify the true shape of the response curve.
**Type 2: Confirm the working range**. The response curve has been mapped previously and is generally understood. Calibrants are measured to determine the specific values on a particular instrument at a particular date. These calibrants are evenly spaced between the LLOQ and ULOL. Any curve that includes a nonlinear response should have sufficient additional points between ULOL and ULOQ to describe the inflection point. Datapoint weighting may be applied to resolve issues of heteroscedasticity. Response values outside of the working range are expected but reported as either below the LLOQ or above the ULOQ.
**Type 3: Confirm the decision point**. Regardless of detector response, there is only a single concentration of interest. Values observed close to that concentration will follow a near linear relationship between concentration and response. Calibration may be a single measurement at the decision point with linear regression to the origin, or a line drawn between the pair of calibrants that bracket the decision point. Observations outside the range of calibrants may have significant error in their quantitated value, but are correctly identified, in a qualitative sense, as above or below the decision point.

For the high-volume laboratory, we presume the Type 1 calibration has been validated and production usage of that assay is now focused on a Type 2 calibration to confirm that the regression remains correct over the working range. Alternatively, the laboratory may perform a Type 2 calibration when the sample provider is only concerned with the ‘actionable’ concentration. When the facility knows the decision point, it may choose to simplify the analytic run and rely on a Type 3 calibration.

### 2.2 Calibration overview

The reported measurement from an analytical instrument is correlated to the amount of analyte being measured, either as a linear (*e.g.*, Beer-Lambert) or a non-linear (*e.g.*, Michaelis-Menten) response. In order to establish the specific correlation between concentration and response, a set of standard samples with known concentrations are measured, plotted, and reduced to a single equation. That equation is then used to back calculate the concentrations of samples, based on the measured response.

The introduction of error into the measurement (see [16] for a review of causes and solutions) creates the risk of leverage or bias in the calculated curve, even without the presence of an outlier. Leverage is the dominant concern for standard samples near the ends of a calibration curve, where any sample error has a disproportionate effect on the curve. Such effects are minimized at the median calibrant concentration, which should, therefore, be near the typical concentration of the unknown samples. When the error is heteroscedastic, as is the case for LC-MS/MS measurements [17], 1/X^2^ weighting of the response can reduce leverage effects [18]. For points near the median calibrant concentration, bias – which only affects the axis intercept – is the dominant concern. To minimize the effect from both concerns, outlier detection and removal is specifically described in the guidance documentation (Table 1).

### 2.3 Studentized deleted residuals

The studentized deleted residual (SDR) extends the concepts associated with the t-statistic into an estimation of scale useful for outlier detection [19]. Briefly, the hat matrix is used as an orthogonal projection of the data to normalize the variance of the residuals. Unlike simple R^2^ calculations, which reduce the measure of variance to a single number between zero and one, the target value for the SDR is dependent on the number of data points, the equation of fit, and the permissible variance. For the seven point linear calibration curves discussed in this work, that target value is 3.68; a calibrant is considered an outlier when the absolute value of the studentized residual for that measurement exceeds the target value.

### 2.4 Experimental data

Experimental data was collected from a longitudinal study across multiple production assays. All regression curves were scaled such that the maximum nominal concentration was 100; the values for each regression coefficient were scaled accordingly. This approach allowed the data quality to be monitored in a neutral fashion, with the characterization of 138 unique calibrant intervals. These intervals were applied to a linear, quadratic, or cubic calibration curve in 54%, 42%, and 4% of the studied methods, respectively. The median number of calibrators was 7, with as few as 1 and as many as 39, for a given compound.

### 2.5 Simulations

All calculations were performed using the R statistical programming language. Two sets of simulated calibration curves were generated, each with seven nominal values. Calibration curves using an equidistant (0, 1, 20, 40, 60, 80, 100) scale and a logarithmic (0, 1, 2.5, 5, 10, 50, 100) scale were established with the same blank, LLOQ, and ULOQ. Response and concentration are in arbitrary units, with response that assumed linear, quadratic, or cubic data. Response was set to the nominal concentration when calculating linear calibration curves, or modified by the higher order coefficients −1e−4 and +1e−7 for the quadratic and cubic terms, respectively. These coefficients correspond to the normalized values seen in laboratory practice. To avoid instability when calculating the SDR, and to replicate the variance effects when the compound peak is divided by the internal standard, a negligible measure of heteroscedastic noise was added to each response value so that the coefficient of variation was 0.001. This corresponds to a tightly controlled response factor. After applying noise, any negative response values were set to zero prior to the regression calculation. USFDA proposed guidance recommends dropping a calibrant when its back-calculated concentration is more than ±15% from the expected nominal concentration, or ±20% for the LLOQ. Each of the seven nominal responses was converted into an outlier by modifying that response to be 16% above or below the expected value. The calibrant at zero concentration (the blank standard) was presumed to remain below the limit of detection, effectively having no error in its measurement.

For each outlier calibrant, one thousand data sets were generated by applying the heteroscedastic noise on the remaining calibrants, and linear regression calculations were run either unweighted or with 1/X^2^ weighting of the data. This procedure was repeated with the outlier value set at ±14% to be just within tolerance, and again at ±21% to observe more significant error where the LLOQ outlier should also be detected. Finally, we ran simulations, assuming a linear response and 1/X^2^ weighting, using equidistant spacing and a single outlier at either ±16% or ±21%, but with all measurements done in triplicate and applying noise with a coefficient of variation of either 0.05 or 0.10 on the non-outlier values to approximate realistic, noisier experimental data.

Within each of the sets, we determined which back-calculated responses were beyond the USFDA threshold limit. We also calculated the SDR for each simulation to determine when the response could be seen as an outlier. It is important to note that the back-calculated concentration was checked for each calibrant on the curve, not merely the known outlier. It was, therefore, possible for an otherwise correct calibrant to be labeled an outlier if the calibration curve was sufficiently skewed.

### 2.6 Schema to display results

**Scheme 2:**
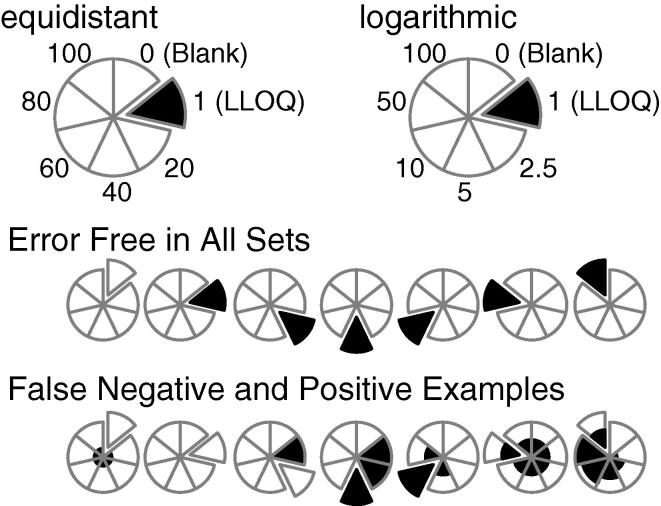
Display of true and false outlier detection.

These simulations produce true positives and negatives – where the outlier is flagged, or the unmanipulated calibrant is not – as well as false positives and negatives – where the unmanipulated calibrant is deemed an outlier, or the actual outlier is not detected. To simplify how these results are reported, we adopted the visualization shown in Scheme 2. Each calibrant is assigned to one of seven wedges of the pie in a clockwise fashion, with the expected nominal concentration following either the equidistant or the logarithmic scale. The blank is included since it is one of the seven points of the curve, and provides a means to observe false positives resulting from the heteroscedastic noise.

For each set of 1000 simulations, wedges are filled according to the fraction of positive identification. The spiked outlier is identified as the exploded wedge, so a row of seven circles will show all simulations under a given condition where each calibrant is made the outlier in turn. The blank will never generate a positive outlier according to USFDA guidance, since it is not possible to show a 15% deviation from the nominal of zero. However, the blank may show positive outliers according to SDR since that calculation measures the degree by which regression aligns with each data point.

A perfectly identified set of outliers is shown in the second row of Scheme 2, where each of the non-blank exploded wedges are completely filled (true positive) and the remaining wedges are completely unfilled (true negative). The third row shows examples where the spiked outlier results in some number of false positives and false negatives. The first circle of this row shows the blank unidentified, as expected, but each of the remaining calibrants produces a false positive 25% of the time. The seventh circle of this row shows the spike on the ULOQ with 50% false negative, and the prior calibrants at 100%, 80%, 60%, 40% and 20% false positive. The remaining five circles show additional examples of false positive and negative results.

## 3 Results and discussion

### 3.1 Justifying a seven-point calibration curve

This proposed justification for the proscribed minimum number of points is based on commonly accepted confidence limits for a normally distributed measurement, and a proof against conventional wisdom for the 3σ edit. Assuming normal distribution of error on any measurement, 95% of all observations will be within 2σ of the mean, and 99.7% will be within 3σ of the mean. This relatively high percentage leads to the common misconception that outliers can be eliminated if the observation is more than three standard deviations away from the mean, even though the very definition of a normal distribution requires three out of every thousand measures to be outside a 3σ boundary. Furthermore, the assertion made by Maronna, Martin, and Yohai [20] is that outlier elimination based only on the normal distribution of measurements is futile. In our proof of that assertion, given the t-statistic

(1) $${\text{t}}_{\text{i}}=\frac{\left|{\text{x}}_{\text{i}}-\overset{¯}{\text{x}}\right|}{\text{s}}\text{,}$$

where *x_i_* is a measured value, $\overset{¯}{x}$, is the arithmetic average, and *s* is the experimental standard deviation with *N* − 1 degrees of freedom, let $x_{1}=c$ and all other values of $x_{2\ldots N}=0$. From this,

(2) $$\overset{¯}{\text{x}}=\frac{1}{\text{N}}\underset{\text{i}}{\sum }{\text{x}}_{\text{i}}=\frac{\text{c}}{\text{N}}$$

and

(3) $$\begin{array}{cc} & {\text{s}}^{2}=\frac{1}{\text{N}-1}\underset{\text{i}}{\sum }{\left({\text{x}}_{\text{i}}-\overset{¯}{\text{x}}\right)}^{2}=\frac{1}{\text{N}-1}\left[{\text{c}}^{2}{\left(\frac{\text{N}-1}{\text{N}}\right)}^{2}+\frac{(\text{N}-1){\text{c}}^{2}}{{\text{N}}^{2}}\right]=\frac{{\text{c}}^{2}}{\text{N}}\\ & \text{s}=\frac{\text{c}}{\sqrt{\text{N}}}\text{.} \end{array}$$

Inserting these relationships for the average and standard deviation into Eq. (1) yields

(4) $${\text{t}}_{1}=\frac{{\text{x}}_{1}-\overset{¯}{\text{x}}}{\text{s}}=\frac{\text{c}\left(\frac{\text{N}-1}{\text{N}}\right)}{\frac{\text{c}}{\sqrt{\text{N}}}}=\frac{\text{N}-1}{\sqrt{\text{N}}}\text{,}$$

which, counter-intuitively, produces a t-statistic independent of the measured value *c*.

The t-distribution is used to mimic the normal distribution under circumstances where the sample size is small and the standard deviation is unknown. An otherwise perfect dataset for an *N* point calibration curve with one infinitely large outlier will produce a t-value equivalent to the maximal standard deviation of the curve, which is the relationship given in Eq. (4). A calibration curve using four data points, for example, will result in a maximum t-value of 1.5 for an outlier. This is equivalent to saying the outlier can be no more than one and a half ‘standard deviations’ from the mean.

Using Eq. (4) to calculate *t* for varying values of *N* shows it will take twelve data points before the maximum t-value exceeds three ‘standard deviations’ and seven data points before exceeding two. Seven measurements before permitting a 2σ elimination aligns with the general tendency to select 95% confidence limits for other aspects of data collection. The need for twelve measurements on the curve before justifying a single 3σ elimination is especially critical to keep in mind when working from empirical rule sets that monitor data quality. It is also worth noting that the above derivation is a worst-case scenario, making no assumptions about prior knowledge of the system such as response linearity or the degree of accuracy and precision on a measurement, and highlights the difficulty associated with outlier identification.

### 3.2 Longitudinal variance in calibration

**Fig. 1:**
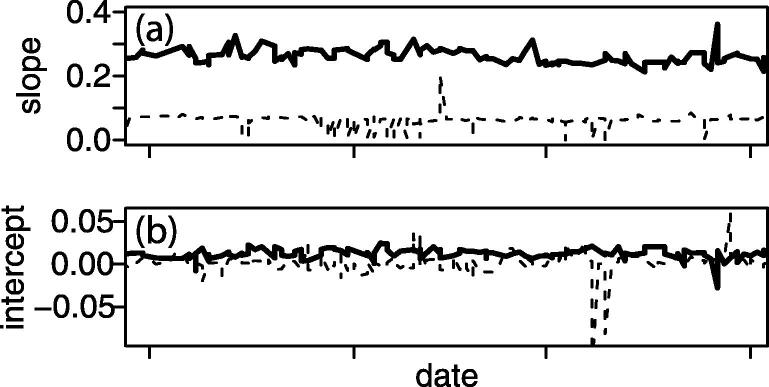
Trendline for slope (a) and intercept (b) of the regression curve for two compounds (solid and dashed line) over a three-month timeframe.

Recognizing that USFDA assay validation requires calibration stability to be measured for a minimum of six runs over several days, we chose to expand our view to a longer timeframe in order to gauge instrument stability in a production setting. Measured over a three-month period, two relatively stable compounds (Fig. 1) generated a median slope of 0.256 and 0.066, and median intercept of 0.0113 and 0.0011, respectively. Both compounds were monitored with a seven-point linear calibration curve, the dashed line corresponding to a compound with roughly equidistant spacing of calibrants (0, 0.5, 1, 10, 40, 70, 100) and the solid line for one with a more logarithmic spacing (0, 0.2, 1.5, 5, 25, 50, 100). The coefficient of variation for each calibrant is between 2% and 16%. The risk from this variability affects both leverage and bias, observed by marked deviation from the median value for the slope and intercept, and should be corrected by outlier removal.

The vast majority of the observed production assays collect a new calibration curve for each batch of data, using a single measure at each calibrant concentration. Collecting, qualifying, and referencing a new curve independent of the data collection frequency is contrary to the expected assay stability required during method validation. It also blurs the purpose of the calibration curve with the purpose of a QC sample. The curve should be built to associate a response to a concentration, and the QC then used to confirm the response associated with a concentration. Collecting a new curve with every batch leads to a scenario where the calibration is being used to confirm the response both before, and instead of, confirmation with the QC samples. The decision to measure in singlicate regardless of calibrant CV values also raises the question of how an outlier might be identified. Recognizing the industry preference to measure a new curve with every batch, it becomes critical to establish a successful approach for outlier detection.

### 3.3 Equidistant versus logarithmic calibrant spacing

Equidistant spacing of calibrants across the working range is the generally accepted method of building a calibration curve. It provides a consistent density of measurements between the LLOQ and ULOQ, and anticipates the analytic run will report values across the entire working range. In practice, we have observed that high-volume LC–MS/MS assays are almost exclusively monitoring the working range with serially diluted standards, clustering measurements at the lower end of the calibration curve. All 138 of the calibration intervals have at least some logarithmic component to their nominal expectation, and only 41 out of 138 have a calibrator between nominal 50 and 100. The median calibrant value is nominal 5, creating a significant skew towards the LLOQ when observing corrective pressure due to bias and leverage.

Choosing the logarithmic scale may be a conflation of purpose between detector mapping (to find the LLOQ) and range confirmation (to verify stability). If so, detector mapping continues to be performed instead of range confirmation even after assay validation. It may also be intentional when analytes have a decision point near the LLOQ. Calibrant clustering would improve curve accuracy near the lower limit, but comes at the cost of relying upon a single measurement for the upper half of the working range.

**Fig. 2:**
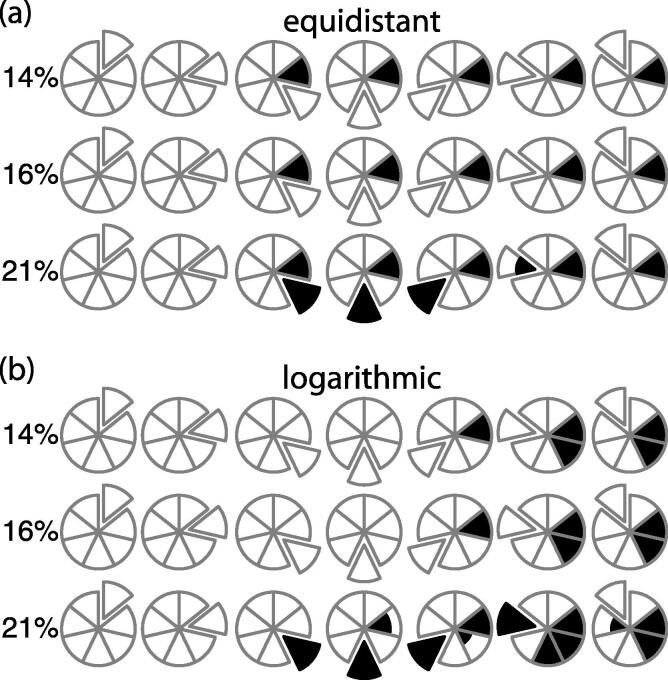
Outlier detection using the back-calculation cutoff with an unweighted linear regression. Spiked outlier performed at three different concentrations shown for the equidistant (a) and logarithmic (b) spaced calibrants. The true outlier is undetected unless it significantly exceeds the back-calculation cutoff.

Correct outlier classification of the LLOQ calibrant is practically impossible when using unweighted regression. Here, we follow the USFDA proposed guidance by calculating the curve using all observations, then back calculating the concentration from the observed response, while considering the calibrant an outlier if this back calculation surpasses the USFDA cutoff. When considering the simulations with equidistant spacing (Fig. 2a), the LLOQ is a false positive at each nominal concentration above the LLOQ, regardless of whether the spiked outlier was below the USFDA cutoff of 14%, above the cutoff at 16%, or above the LLOQ cutoff at 21%. Frustratingly, the LLOQ was a false negative with 21% spiked error, a pattern that continues with the logarithmic calibrant spacing (see below). Only in the ‘spiked blank’ or an LLOQ spike below the 20% threshold does the LLOQ correctly identify as a true negative.

The equidistant scale of calibrants has additional and worrisome issues with the spiked outliers above the LLOQ. The 14% spike is an understandably true negative, being below the 15% threshold. However, the 16% spike shows false negatives in every case, and the highest two calibrants are false negative even when they are subjected to a 21% spike on their nominal concentration. These last two are particularly notable, since they show the effect of leverage on the curve, skewing the slope to such an extent that this 21% actual error appears to be within the 15% tolerance.

Moving to a logarithmic scale provides additional low concentration calibrants to the curve, but does not eliminate the false identification problems for the LLOQ (Fig. 2b). To the contrary, outlier calibrants near the ULOQ cause additional false positives compared to the equidistant scale. Depending on the size of the outlier spike, an outlier at the log-scale nominal 50 and 100 will cause calibrants at nominal 2.5 and 5 to become false positives. Even a 14% spike on the nominal 50 calibrant, a value that should be considered within tolerance, will create the appearance of an outlier at the two lowest calibrants. A 21% spike on the nominal 50 does flag as a true positive, but also adds the nominal 5 as another false positive. Four of the seven points on this curve would be rejected due to a single outlier. The net result of unweighted curve fitting is to throw away correct points in exchange for keeping the incorrect ones.

**Fig. 3:**
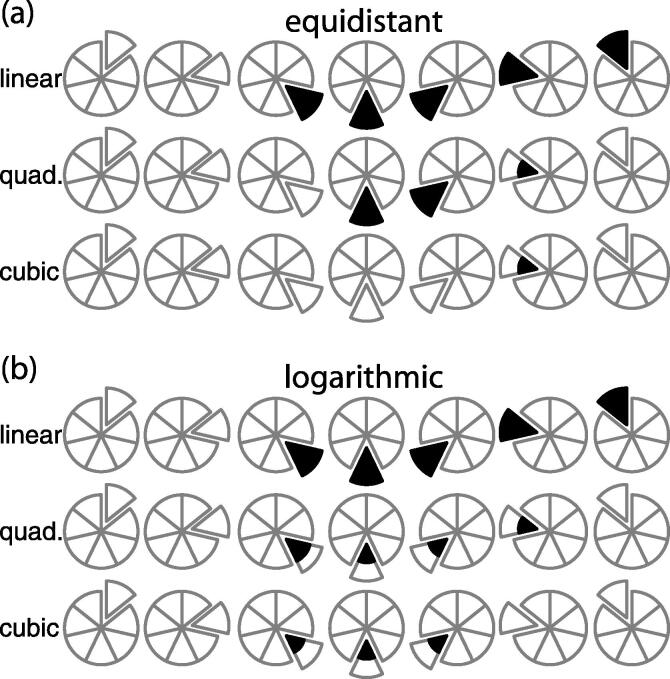
Outlier detection using the back-calculation cutoff with 1/X^2^ weighted data, the spiked outlier at ±21% of nominal, and equidistant (a) or logarithmic (b) spacing of calibrants using a linear, quadratic or cubic dataset.

These false positive issues are eliminated when applying 1/X^2^ weighting to the points during the curve fit, but the false negative outlier calibrants remain until the error is well above the cutoff threshold. This weighting factor is the most appropriate method for resolving the heteroscedastic data generated with LC–MS/MS instrumentation [17], and the simulations confirm that for both equidistant and logarithmic spacing of calibrations, there are no false positive calibrants when the outlier spike is 14%, 16% or 21% of the expected value. While the 21% spike showed the true positive for all but the LLOQ (Fig. 3, rows marked linear), the 16% spike outlier was a false negative for all calibrants (not shown). The guidance for finding an outlier is not strictly attainable unless the actual error is well above the expected threshold.

### 3.4 Quadratic and cubic regression data

A common industrial practice when measuring metabolites and other small molecules is to “stretch the curve” out of the linear response region in order to report a high quantitative value instead of requiring sample dilution and reinjection. A quadratic or cubic fit is applied to the data with an implicit assumption these higher order terms will be small enough to keep the linear portion of the curve essentially linear, while including the necessary curvature at the ULOQ. However, outlier detection is significantly poorer with these higher order fits than with the linear case. Using 1/X^2^ weighting on the linear, quadratic, or cubic datasets fails to find any outlier with the 16% spike in either spacing system. Although false positives remain absent with the 21% outlier spike (Fig. 3), false negatives continue to be problematic.

Progressively higher order equations of fit show an increased rate of false negatives. The cubic data with equidistant spacing is only able to identify one calibrant in half the cases, while all other spikes are reported as false negatives. The LLOQ continues to always appear as a false negative, and the ULOQ is a false negative for any curve more complex than linear. This is completely contrary to the desired intent of extending the range, since inaccuracies outside of the linear region are undetectable. Furthermore, an outlier at the extremes of the working range can significantly skew the calibration curve, and higher order regression curves favor deviation from the correct values instead of identifying the outlier. Most problematic are outliers at the ULOQ, where the outlier can produce a significant arc on the regression line and force back calculation to report either zero or two concentrations for a given response.

### 3.5 Back calculated deviation using SDR

USFDA regulations stipulate that removal of an outlier should be based only on the back-calculated concentration. Simple back calculation relies on a regression constructed with all calibrants. This necessarily, and sometimes severely, skews the curve to favor inclusion of any outliers in that curve. The hat matrix used in the SDR allows for a single calculation to check each calibrant against a model built without that particular point. Since the SDR does not use a nominal percentage cutoff, it detects outliers regardless of scale, calibrant weighting, or regression curve type.

When calculating the SDR on a 1/X^2^ weighted calibration curve, the true positives were always found for both equidistant and log spacing of the calibrants at 14%, 16%, and 21% (Supplemental Fig. 1). When working with the logarithmic scale, false positives at the two largest calibrants using the quadratic and cubic datasets warrant particular discussion. Because the outlier is defined only by the SDR, the mathematical model cannot distinguish between a spike on nominal 50 with a correct nominal 100, or a correct 50 with a spiked 100. Checking either calibrant returns a calculated SDR that exceeds the target threshold, giving correct identification of the true outlier, but also incorrectly identifying the other calibrant as an outlier. The net result is that, in the logarithmic spacing scenario with quadratic or cubic data, an outlier on either of the highest two calibrants will tend to exclude both calibrants and reduce the working range into the linear region.

While SDR is a robust means of finding outliers, regulations are focused on the percent deviation from nominal and so the back calculation must still be performed. Once an SDR-flagged calibrant is detected, the concentration can be back calculated using a regression curve built from the remaining *N* − 1 data points, and rejected only if it exceeds the deviation threshold. Following this two-stage process successfully classifies all equidistant calibrants. The logarithmic calibrants are also improved, but the ULOQ remains classified as a false positive when the actual outlier is the nominal 50. When the true outlier (nominal 50) is dropped and back calculated to a curve using only correct values, that outlier is correctly identified. However, when the false outlier (nominal 100) is dropped and the true outlier is kept, the back calculation is performed referencing a regression curve built using an outlier concentration, and now labels the correct point as if it exceeded the reporting threshold. Without additional sampling at the upper end of the working range, higher order regression on the logarithmic scale remains susceptible to false positives and reduction of the working range.

### 3.6 Comparative outlier methods with triplicate measurement

**Fig. 4:**
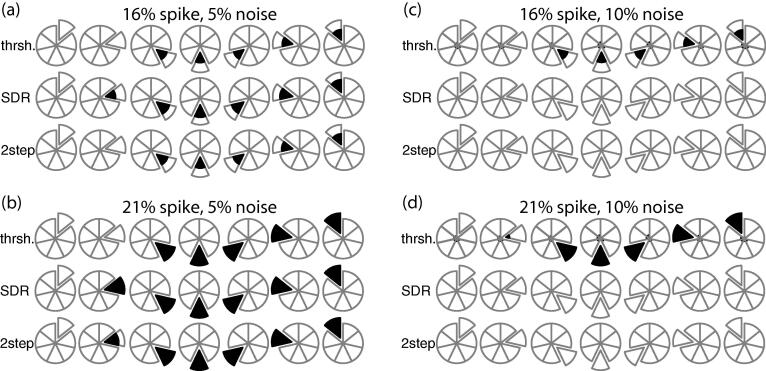
Outlier detection with triplicate measurement at each concentration on a linear dataset with equidistant calibrant spacing and 1/X^2^ weighted data. The relative noise applied to non-outlier data points was 0.05 (a, b) or 0.10 (c, d) and the outlier spike was either 16% (a, c) or 21% (b, d). The upper, central, and lower row of each subfigure correspond to detection using the back-calculation threshold, the SDR, or the SDR followed with confirmation by back calculation, respectively.

We next examined a more realistic context on a linear curve with 1/X^2^ weighting by applying both a greater degree of error and triplicate measurement of the calibrants. With 5% (Fig. 4a, b) and 10% (Fig. 4c, d) normalized heteroscedastic noise on the non-outlier measurements and a 21% noise spike, standard back calculation identifies all spiked outliers above the LLOQ. However, a 16% spike and standard back calculation results in roughly half of the outliers being seen as false negative. The LLOQ outlier remains undetected even with the triplicate measurement. The SDR, alone (middle row of Fig. 4a-d), increases the true positive rate with 5% noise such that the LLOQ is now found, but converts every calibrant into a false negative at the 10% noise level. Combining the SDR for outlier detection followed by the threshold check using back calculation (bottom row of Fig. 4a-d) increases the false negative rate at the LLOQ, and otherwise maintains the true and false positive rates seen with SDR alone.

Critically, the triplicate measurement does not prevent false positive identification using standard back calculation. There are an average of 3 false positives per 1000 simulations at 5% heteroscedastic noise, which increases to 120 per 1000 at 10% noise. Moving to the SDR method of outlier detection brings that false positive rate to less than one tenth of the applied noise in all cases, which is maintained when the SDR detection is followed by the threshold check.

**Fig. 5:**
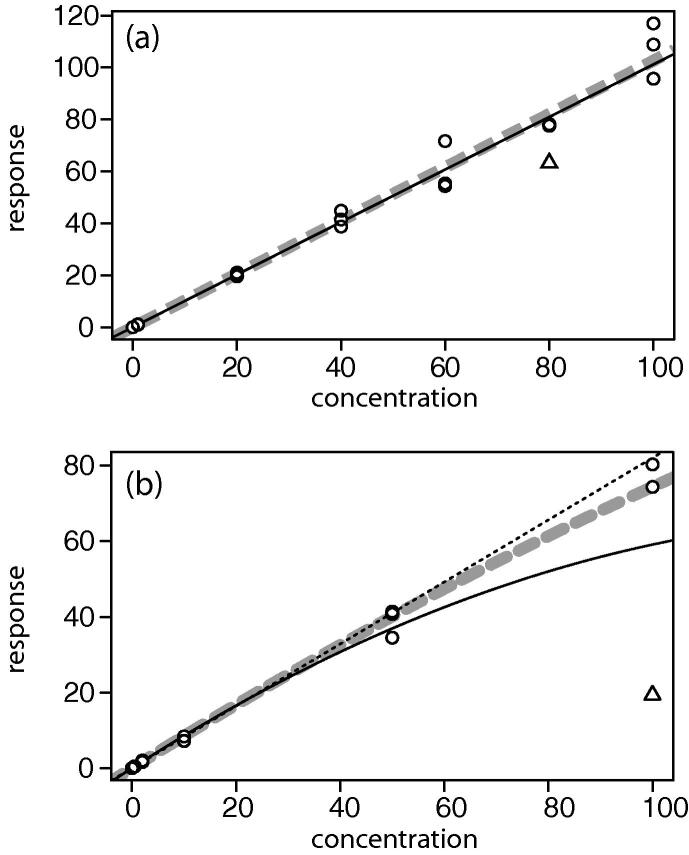
Regression curves (○) with known outlier (△). Simulation of triplicate data (a) with 10% error on all points and a 21% spike on the nominal 80 was not detected by the combined technique, but the difference in regression with and without the outlier (solid and dashed line) is negligible. Triplicate experimental data using a quadratic fit (b) was normalized and identifies a single outlier at nominal 100 using the combined outlier technique. A linear fit excluding the outlier is shown as a dotted line, for reference.

The false negative outliers highlight how noise in the system is equivalent to outlier inclusion. A typical false negative example is shown in Fig. 5a, with a −21% outlier drawn as a triangle at nominal 80. The regression curve calculated with and without that outlier is drawn with the solid and dashed line, respectively, and is negligibly different. Indeed, the randomly created outliers at nominal 60 and 100 are as visually significant as the spike. The fact that the regression line does not change with outlier inclusion helps explain the difficulty in labeling the outlier and reinforces how noise effects are minimized by replicate observations. We find the combined technique for outlier detection outperforms current practice, properly accepting an outlier in the context of high noise, and approaching perfect outlier assignment as the measurement noise decreases.

Triplicate measurement alone is not sufficient to resolve potential outliers, especially in the context of logarithmic scaling and higher order regression. As shown in Fig. 5b, this experimental dataset has one apparent outlier at the ULOQ, while the remainder of the noise appears reasonable. For this compound, the intended regression is quadratic with 1/X^2^ weighting. The seven nominal concentrations are logarithmic with values (0, 0.2, 0.5, 2, 10, 50, 100) and the three-month trend in the data informs us the coefficient of variation for this compound is 14%. Applying standard back-calculation techniques would fit all data points, as shown with the solid line. This line classifies seven calibrant measurements as outliers, including all of the nominal 100 and two out of three of the nominal 10. Rejecting seven of twenty-one calibrants is a 33% failure rate, and USFDA proposed guidance would require the run to be resampled. Applying back calculation, based on SDR identification, results in a single outlier, drawn as a triangle in the figure, which is excluded in order to draw the wide dashed line. This combined technique was sufficient to find a clear outlier and to permit the batch to proceed for further analysis.

## 4 Conclusions

Calibration is not a simple matter of comparing responses and concentrations. At a minimum, there is a need to “run the experiment before you run the experiment” to identify the typical response curve on the instrument, and to compare that curve to the boundary conditions for the expected analytic range. Working in a regulated environment brings the additional expectation of a method validation procedure that identifies and then controls the factors responsible for signal variance. For example, approaching this validation as a study of the analysis of variance (ANOVA) allows all mutable components of the system to be examined, from the instrument to the analyte to the process of operation. Once that validation is complete, however, the role of calibration shifts from detector mapping to confirmation of response stability.

Any concern about the robustness of a production system should be focused on stability confirmation. Factors identified as weakly influencing the response do not require significant additional controls within the system, while those with a strong influence will mandate full recharacterization after the failure of the QC samples. Given that many laboratories may not dedicate a single instrument to a single assay, variations in reagent preparation, spectrometer health, and technical expertise can shift the slope of the calibration curve by 10% or more. Furthermore, the analyte may be difficult to measure, or the assay inherently unstable, or the variability poorly characterized during method development. But a process that demonstrates substantial day-to-day variance should not be expected to remain stable over the course of a single analytic run, and a process that is stable for weeks does not require a unique calibration curve for every run. A daily calibration curve to recharacterize the instrument does not resolve the actual variability. Instead, it is an inappropriate soft correction for possible variance where each calibration curve is presumed error-free while simultaneously describing the difference from prior runs.

The common industrial practice of generating a new calibration curve for each analytical run only answers the question, “is this curve good,” when the true question should be, “has the instrument changed.” The typical assignment of a single run to a single 96-well plate means the seven calibrants are measured once because adding calibration replicates to the plate will significantly reduce the number of available unknown samples. As a result of this singlicate measurement, one outlier can drive the reported concentration away from the actual value while remaining hidden as a false negative. Worse, that false negative is capable of producing one or more false positives at the lower end of the working range, causing the run to reject correct values and retain incorrect values when building the curve.

Exclusion is the simplest method to reduce the risk from an outlier. Based on our derivations, seven calibrants is the minimal number of points on a curve, which permits a single exclusion, while maintaining 95% confidence in the accuracy of the curve. It should be stressed that this is 95% confidence in the curve itself, not in the accuracy of any single measurement along the curve. By definition, the lowest and highest calibrants define the LLOQ and ULOQ, but only the Eurachem requirements state the calibrants have to be evenly spaced across the working range. Our simulations reinforce the equidistant approach and demonstrate how logarithmic scaling is susceptible to leverage effects under conditions where the outlier is at the upper end of the curve; an event which can call the upper half of the working range into question. This dilemma is accentuated when the ULOQ is extended into the nonlinear region of the instrument response. Any possible advantage sought by expanding the working region beyond the ULOL is negated by the increased risk of measurement error.

Ultimately, we recommend the following procedure:

1. Begin by mapping the detector using serial dilutions to determine the LLOQ and confirm the stability of the response.
2. Aim to keep all calibrants in the linear region, so that the ULOQ is also the ULOL.
3. Use at least seven calibrants, including a blank and an even distribution between LLOQ and ULOQ, applying 1/X^2^ weighting of the points to fit the curve.
4. Maintain the data weighting and curve-type once the assay is in production.
5. Avoid using R^2^ as a check of instrument stability before reporting results. Instead, apply the combined technique of SDR for outlier detection followed by guidance thresholds to confirm rejection of any potential outlier.
6. Rely on the QC samples and not the standards for information about the analytical run staying within expected variance.

For strongly regulated environments, the requirement to establish a highly stable assay platform provides an opportunity unavailable to the low-volume laboratory. Rather than expend samples to calculate a new calibration curve in each batch, it is more efficient and accurate to collect a separate calibration batch. Replicate measurements of each calibrant in this standards batch would better reveal the presence of outliers and also minimize the effects of non-outlier variability. That curve could then be used for all subsequent runs until the quality control samples within an analytical batch reveal the assay is no longer in control. Only after a QC failure, or some change in a factor with strong influence, should a new calibration batch be collected. Guidance documentation requires only a small set of QC samples to be included with each analytical batch, allowing more subject samples to be measured during a production run. Remaining concerns about assay stability are best addressed with QC replicates, within the batch, instead of a curve measured in singlicate. This approach has already been adopted by a number of laboratories.

## Funding

The authors are salaried employees of Indigo BioAutomation that produces mass spectrometry quantitative analysis software including methods for calibration. The research presented here was funded as part of Indigo’s ongoing research into improved methods for all aspects of quantification.
