# Supplementary material for: A proposal to improve calibration and outlier detection in high-throughput mass spectrometry
Source: Clin Mass Spectrom. 2017 Jan 3;2:25–33. doi: 10.1016/j.clinms.2016.12.003 (PMC11322755; doi:10.1016/j.clinms.2016.12.003)
Supplement: Supplementary data 1 [file mmc1.doc]

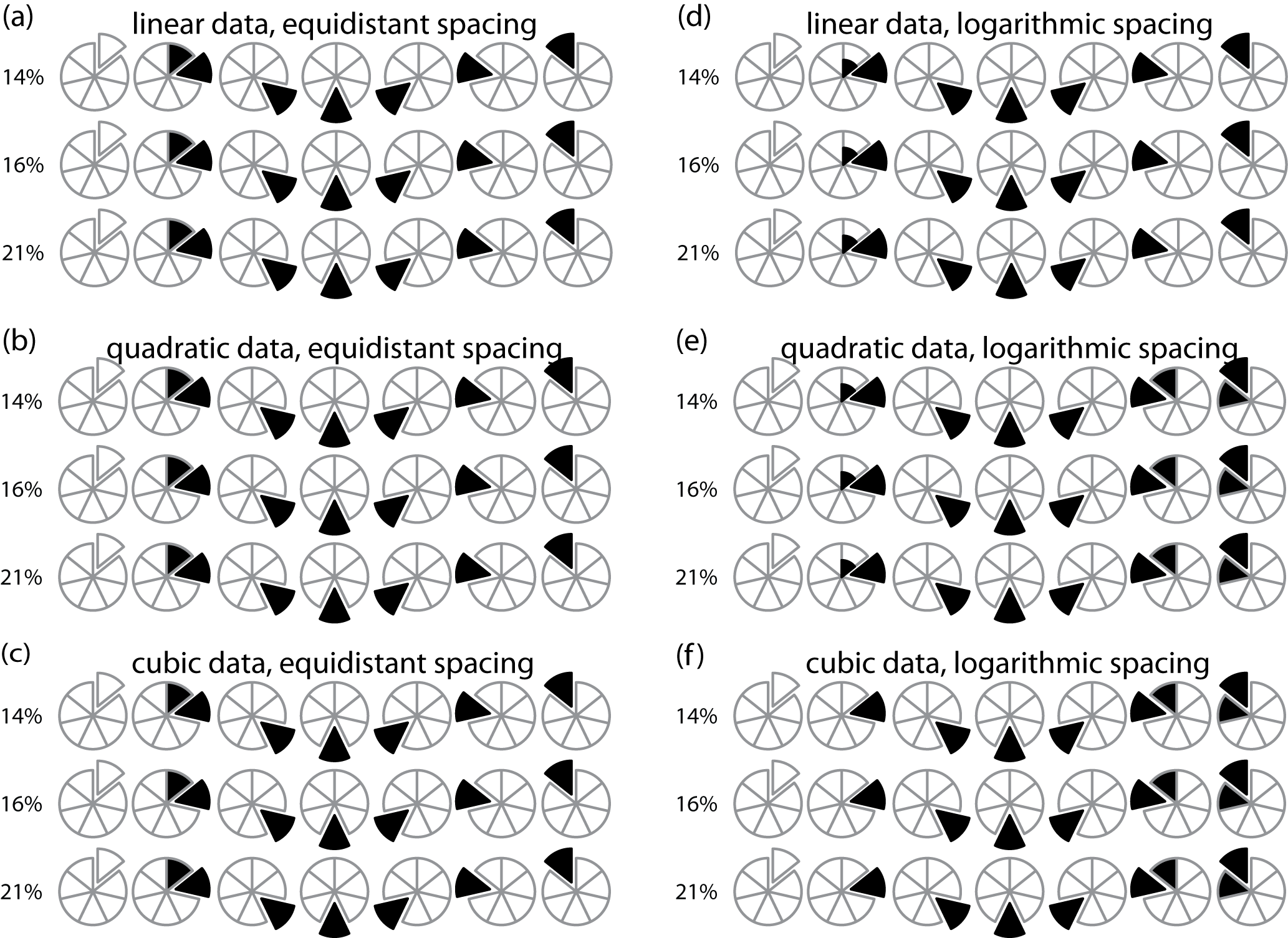


Supplemental Figure 1. Outlier detection using only the studentized deleted residuals. A 14%, 16%, or 21% spike on linear (a, d) quadratic (b, e) or cubic (c, f) data is shown. Linear regression was done with 1/X^2^ weighting, with equidistant (a, b, c) or logarithmic (d, e, f) spacing.
